# The ‘hand-brain-emotion’ axis: a theoretical model for handicraft art-based intervention in anxiety disorders through neuroplasticity and autonomic regulation

**DOI:** 10.3389/fpsyg.2026.1786210

**Published:** 2026-04-09

**Authors:** Ruiqiao Guan, Wenxiao Wang, Jiaming Dai, Dengdeng Li

**Affiliations:** 1 Department of Chinese Medicine, The First Affiliated Hospital of Dalian Medical University, Dalian, Liaoning, China; 2 Faculty of Humanities and Arts, Macau University of Science and Technology, Taipa, Macao SAR, China

**Keywords:** anxiety disorder, hand-brain-emotion axis, handicraft, neuroplasticity, non-pharmaceutical treatment

## Abstract

This review proposes a theoretical model, the hand-brain-emotion axis, which is based on the neuro psychological theory of how handicraft art activate specific brain regions to enhance neuroplasticity and emotional regulation. Anxiety disorders are common mental health disorders that have a profoundly impact on patients’ quality of life. Current pharmacological treatments, while effective to some extent, are limited in efficacy and often accompanied by side effects. Against this backdrop, exploring non-pharmacological interventions has become crucial. Research has demonstrated that handicraft art activities can substantially attenuates anxiety levels and promote the secretion of neurotransmitters, thereby improving mental health. This review aims to provide a theoretical foundation for future clinical designs of diverse handicraft art programs to improve the emotional and cognitive functions of anxiety disorder patients. It also offers theoretical support for further exploring the dynamic effects of handicraft art activities on the brain using techniques such as neuroimaging and neuroelectrophysiology. The hand-brain-emotion axis theory presented in this review provides a solid theoretical basis for the application of handicraft art activities as a novel therapeutic approach for anxiety disorders. It is also expected to promote innovation and development in the fields of neuropsychological health and art therapy.

## 1 Introduction

Anxiety disorder (AD), commonly referred to as anxiety neurosis, is a prevalent mental health issue in contemporary society. It includes several types, such as generalized anxiety disorder, panic disorder, social anxiety disorder, separation anxiety disorder, and anxiety related to physical health issues (Penninx et al., 2021). Anxiety disorder typically presents as a chronic and fluctuating condition. It is frequently comorbid with other illnesses, and its low rates of recognition and treatment lead to a significant economic burden on patients, families, and society.

At present, there is no targeted medication available for treating anxiety disorders. Common clinical medications for anxiety include selective serotonin reuptake inhibitors (SSRIs) like fluoxetine and citalopram, serotonin-norepinephrine reuptake inhibitors such as venlafaxine, and benzodiazepines like diazepam. While traditional pharmacological treatments for anxiety disorders have demonstrated some efficacy, they are associated with numerous limitations (O’Leary and Khan, 2024). These include poor patient tolerability, high risk of dependency, and potential side effects such as dizziness, headache, and memory impairment (O’Leary and Khan, 2024). This has prompted the medical community to continuously explore safer and more patient-friendly non-pharmacological alternative therapies. In recent years, non-pharmacological interventions have gradually gained prominence. Among them, art therapy has emerged as a field of significant interest. By leveraging creative artistic activities to enhance an individual’s psychological state without reliance on chemical substances, art therapy offers a novel approach to the treatment of anxiety disorders.

Handicraft art therapy has emerged as a novel complementary treatment method, garnering increasing interest from both clinical practitioners and academic investigators. Handicraft-based interventions, including pottery, embroidery, knitting, and woodworking, demonstrate beneficial effects on anxiety within occupational therapy settings. From a neuroscientific standpoint, the underlying mechanisms involve sensorimotor integration and modulation of reward pathways in the brain. These activities engage fine motor skills and bilateral coordination, occupying resources within the sensorimotor network. Research indicates that during anxious states, this network shifts from specialized processing to a more globally integrated mode, redirecting attention away from internal anxiety-related cues and altering functional connectivity in emotion-regulating regions such as the amygdala and prefrontal cortex (Su et al., 2026; Wang et al., 2025). Concurrently, handicraft activities offer immediate sensory satisfaction and a sense of achievement upon task completion, which activates reward circuits including the mesolimbic dopamine system. Prior studies have shown that the medial prefrontal cortex (mPFC) modulates reward-seeking behaviors and emotional reactions to reward deprivation through dopamine D2 and NMDA receptors, contributing to anxiety reduction (Birnie et al., 2020; de Oliveira Lima et al., 2026).

Handicraft art therapy has the potential to regulate neurobiological mechanisms. Engaging in handicraft activities enables individuals to concentrate on a particular creative process. This ‘flow’ state can effectively reduce anxiety levels and promote the secretion of pleasure-associated neurotransmitters in the brain (Dieterich-Hartwell, 2023). Additionally, handicraft activities are believed to enhance the physiological foundations of mental health and restore balance to the endocrine system, thereby alleviating anxiety symptoms (Leckey, 2011). In summary, handicraft art therapy is an innovative approach that combines medicine and art to treat anxiety and somatic functional disorders, warranting further research and clinical application. In this review, we will focus on how handicraft art activities regulate anxiety-related brain networks (such as the amygdala and prefrontal cortex) through mechanisms like sensorimotor integration, attention shifting, and reward pathway activation, thereby proposing the underlying ‘hand-brain-emotion’ axis theory and exploring its application prospects in clinical occupational therapy.

## 2 How anxiety disorders affect the brain’s structure

The brain structure and function in individuals with anxiety and depressive disorders exhibit distinct characteristics compared to those in healthy populations. In neuropsychology, the frontal lobe is integral to cognitive processes, with its biological activities shaping human values, moral reasoning, emotional responses, and personality traits research has demonstrated that during resting states, patients with depression display markedly elevated activity in the ventromedial prefrontal cortex (vmPFC) (Mackey and Petrides, 2014; Ongür et al., 2003). Furthermore, increased activation in the dorsolateral prefrontal cortex (dlPFC) has been linked to depressive symptoms. Anxiety disorders predominantly involve alterations in the hippocampus, prefrontal cortex, and amygdala. Notably, individuals with social anxiety disorder (SAD) show elevated activity in the insular cortex under social stress conditions, while activity in the ventral striatum is reduced (Koenigs and Grafman, 2009). The section will be elaborated in detail according to the structural characteristics of brain neuroanatomy.

### 2.1 Atrophy of the hippocampus

The hippocampus is a critical brain structure essential for learning and memory processes. Studies indicate that individuals with anxiety disorders frequently display hippocampal atrophy. Research employing magnetic resonance imaging (MRI) has consistently demonstrated that the hippocampal volume in patients with anxiety is markedly reduced compared to healthy controls (Xie et al., 2021). This reduction may be associated with chronic stress and neuroinflammation induced by anxiety, which can result in neuronal damage and cell death (Won and Kim, 2020). Additionally, hippocampal atrophy may impair emotional regulation and memory functions, potentially worsening anxiety symptoms (Teng et al., 2024). Anxiety disorders are prevalent among adolescents, and the immature development of the hippocampus may heighten their vulnerability to anxiety, a conclusion supported by neuroimaging evidence (Xie et al., 2021). Collectively, hippocampal atrophy serves not only as a biological indicator of anxiety disorders but may also play a crucial role in their underlying pathological mechanisms.

### 2.2 Dysfunction of the prefrontal cortex

The prefrontal cortex plays a pivotal role in executive functions, emotional regulation, and decision-making processes. Individuals diagnosed with anxiety disorders frequently exhibit impaired prefrontal cortex activity, especially when processing emotional stimuli (Sylvester et al., 2021). Research utilizing functional magnetic resonance imaging (fMRI) indicates that, in comparison to healthy controls, patients with anxiety disorders demonstrate markedly reduced activation in the prefrontal cortex upon exposure to negative emotional cues. This activation is thought to compromise their capacity for effective emotional regulation (Xie et al., 2021). Additionally, the functional interplay between the prefrontal cortex and the amygdala is disrupted in these patients, potentially resulting in an amplified reaction to perceived threats (Xie et al., 2024). Such neural dysfunction not only influences emotional states but may also impair cognitive performance, thereby exacerbating the symptomatology associated with anxiety disorders (Gorka et al., 2020).

### 2.3 Abnormal activation of the amygdala

The amygdala is a pivotal neural structure involved in the regulation of emotional processing, with a specialized role in mediating fear and anxiety responses. Individuals diagnosed with anxiety disorders often demonstrate elevated amygdala activation, particularly when confronted with threatening cues (Zhu et al., 2023). Studies indicate that exposure to emotionally charged stimuli elicits greater amygdala activity in patients with anxiety disorders compared to healthy controls (Tassone et al., 2024). This heightened activation exhibits a positive correlation with the clinical severity of anxiety symptoms. Furthermore, as previously noted, impaired functional connectivity between the amygdala and the prefrontal cortex is recognized as a key pathophysiological mechanism underlying anxiety disorder. A diminished inhibitory influence from the prefrontal cortex may result in excessive amygdala reactivity, thereby intensifying the manifestation of anxiety symptoms (Shin and Liberzon, 2010).

## 3 How does handicraft art therapy change the brain?—Handicraft activities serve as a way to heal anxiety disorders

### 3.1 Brain regions and artistic creation—the neural mechanisms underlying art production

**Figure 1:**
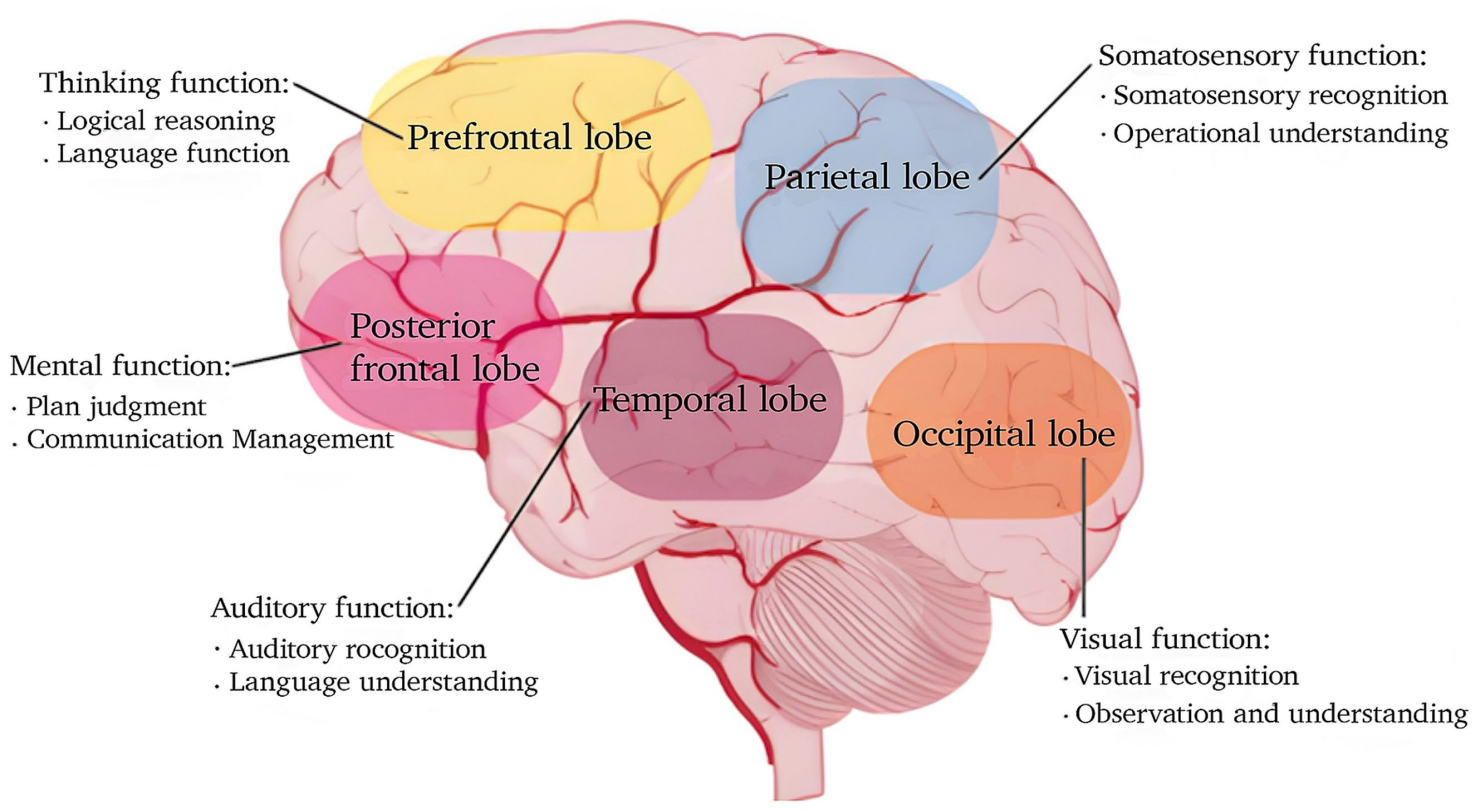
Functions of major brain regions and their roles in artistic creation (original figure created by the authors).

Artistic creation involves the coordinated activity of multiple brain regions. The prefrontal cortex supports higher-order cognitive processes such as planning, decision-making, and creative ideation. In visual art production, the parietal lobe is crucial for spatial awareness and the coordination of bodily movements (Orwig et al., 2021). For musical composition, the temporal lobe is involved in processing both linguistic and musical information, as illustrated in Figure 1 (Barrett et al., 2020).

Neuroimaging methods, including functional magnetic resonance imaging (fMRI) and near-infrared spectroscopy, are frequently used to investigate brain regions linked to creativity and their functional connectivity. Research has further demonstrated that creative activity engages several brain areas, such as the prefrontal cortex, superior temporal gyrus, and hippocampus, which are important for assessing novelty and practical relevance (Ren et al., 2020; Jin et al., 2024). Additionally, creative expression is associated with the dynamic reconfiguration of neural networks. This indicates that creativity relies not only on the activation of specific brain regions but also on the integration among distinct neural circuits (Feng et al., 2019). By analyzing the brain regions activated during artistic tasks could contribute to a better understand of how the brain processes and responds to complex stimuli, offering a neuroscientific rationale for applying handicraft art therapy in the treatment of anxiety disorders and related conditions.

### 3.2 Handicraft art activities boost specific areas of the brain

Handicraft art activities impact brain function and structure through various pathways. Engaging in such tasks requires sustained attention, creative thinking, and problem-solving, which collectively stimulate multiple brain regions, notably the prefrontal and parietal cortices. These areas are integral to cognitive processing, emotional regulation, and social interaction. The complexity of handicraft-based tasks demands high levels of neural coordination, activating specific brain networks involved in fine motor control, visual integration, and creative cognition.

Piper Hutson’s research synthesizes perspectives from neuroscience, psychology, and cultural studies. It demonstrates that engaging in fiber arts activities (such as weaving, sewing, embroidery), which involve following complex patterns, processing spatial relationships, and correcting errors, can effectively activate the prefrontal and parietal cortices associated with problem-solving, decision-making, and sensory integration (Hutson and Hutson, 2024).

This further supports our viewpoint that the cognitive challenges involved in participating in handicraft arts stimulate neuroplasticity in the brain, which refers to the brain’s capacity to reorganize and strengthen neural connections to adapt new tasks.

Furthermore, the fine motor skills required for activities such as threading a needle, demand hand-eye coordination and precise motor control, thus further engaging the sensorimotor cortex.

Electroencephalography (EEG) studies have shown that during handicraft activities engagement, there is a marked rise in midline theta wave (Fmθ) activity within the prefrontal cortex, reflecting a state of relaxed concentration (Shiraiwa et al., 2020). Evidence suggests that handicraft participation can substantially enhance neural activation, promote synaptic connectivity, and improve both cognitive performance and emotional well-being (Ikeda et al., 2022). Furthermore, recent investigations reveal that handicraft activities stimulate the brain’s reward circuitry, leading to the release of neurotransmitters such as dopamine. This process elevates mood and increases motivation, thereby helping to reduce symptoms associated with anxiety and depression (Orui et al., 2024).

### 3.3 Physiological basis of handicraft art therapy: stress hormones and pleasure neurotransmitters

The physiological effects of engaging in handicraft activities are primarily mediated through alterations in stress hormones and neurotransmitter levels. Under conditions of stress, cortisol and adrenaline concentrations typically rise (Hwang et al., 2019). However, participation in handicraft-based tasks has been shown to suppress the secretion of these stress-related hormones. Furthermore, such activities can enhance the synthesis of neurotransmitters, including dopamine and serotonin, which are associated with positive emotional states such as pleasure and well-being. Through involvement in hand-crafting, individuals often attain a sense of achievement and derive enjoyment and relaxation from the process. Research indicates that the repetitive and rhythmic motions involved in fiber art can produce effects akin to mindfulness meditation, stimulating the parasympathetic nervous system and lowering levels of stress hormones. Successfully overcoming challenges in such activities may also engage the brain’s dopamine reward pathways, establishing a beneficial cycle of “effort and reward” (Hutson and Hutson, 2024). Additionally, social environments outside of family and work settings offer individuals a secure and inclusive space for collaboration. Through these interactions, participants can effectively diminish feelings of social isolation while strengthening their sense of belonging and social integration. Collectively, these perspectives imply that handicraft-based activities may possess therapeutic potential for alleviating symptoms of anxiety and depression.

These physiological changes suggest that handicraft activities serve not only as a medium for creative expression but also as a means to support psychological and physiological health at a biological level. Consequently, integrating handicraft-based interventions into therapeutic regimens for individuals with anxiety disorders may effectively contribute to improved mental health outcomes.

### 3.4 Handicraft art therapy: promoting the restructuring of brain neural connections

**Figure 2:**
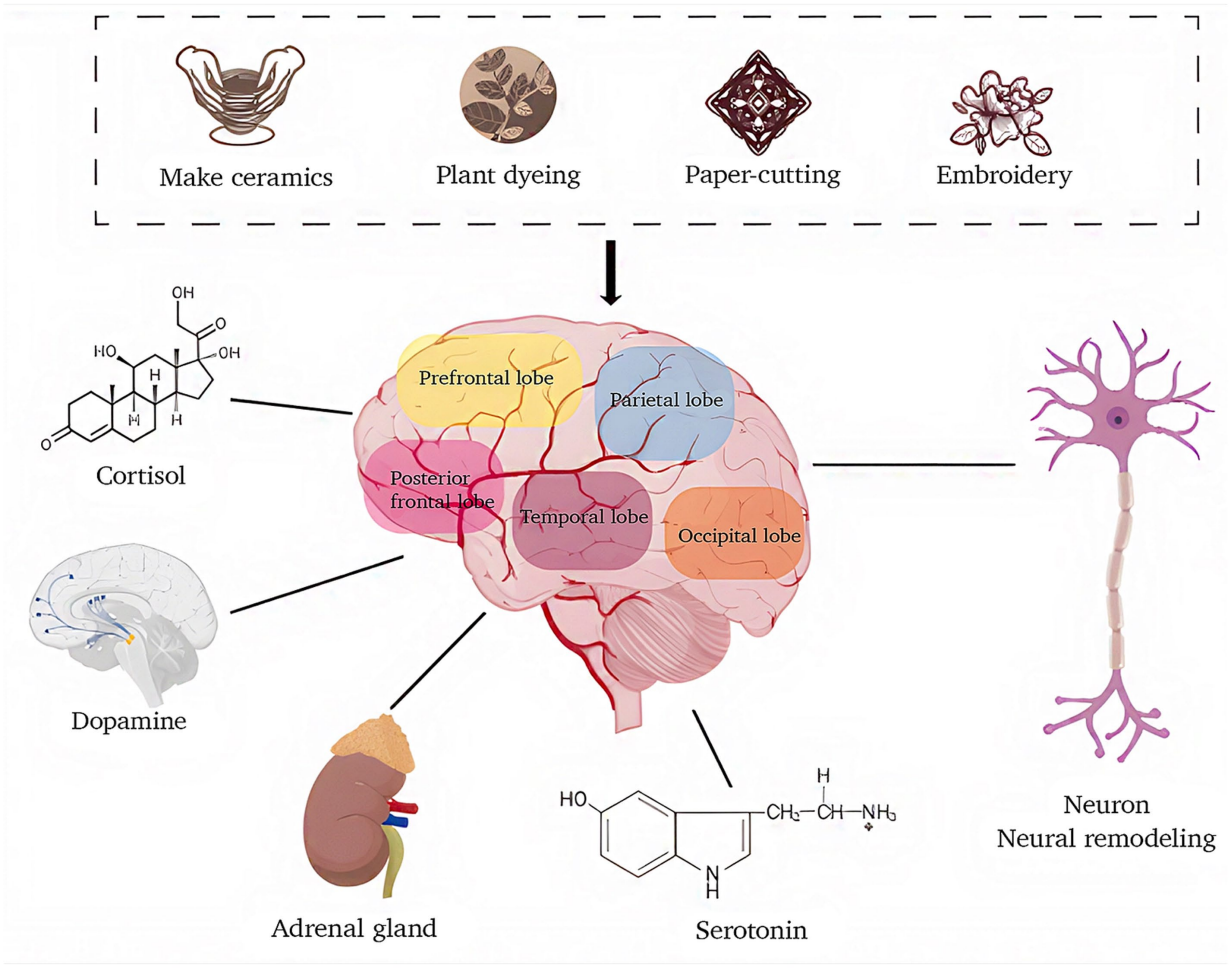
Mechanism diagram of handicraft art therapy alleviates anxiety by regulating brain hormones and restructuring brain neural connections (original figure created by the authors).

Neuroplasticity denotes the capacity of the nervous system to modify its structural and functional properties in response to learning and experiential input. Handicraft-based interventions are strongly associated with neuroplastic mechanisms. Participation in such activities enables individuals to refine their manual dexterity, which subsequently fosters the formation and reorganization of neural networks within the brain. For instance, research indicates that individuals engaged in handicraft tasks demonstrate enhanced white matter integrity in specific cerebral regions following task completion, potentially leading to improvements in motor performance and visual decision-making processes (Lewandowska et al., 2022). Additionally, handicraft activities can strengthen synaptic linkages among neurons, facilitating long-term potentiation (LTP) and thereby augmenting learning and memory functions. This neurobiological process is closely tied to the role of glial cells, including astrocytes, which are essential for modulating neuronal activity and underpinning neuroplastic adaptations (Hughes, 2021). Consequently, integrating handicraft activities into therapeutic regimens for anxiety disorders not only offers an enjoyable experience but also substantially contributes to the restoration of brain function and the enhancement of cognitive abilities. The schematic diagram is shown in Figure 2.

## 4 Psychological mechanisms of handicraft art therapy

### 4.1 Creative expression and embodied cognition

The psychological underpinnings of handicraft therapy are rooted in psychoanalytic theory, the facilitation of embodied cognition through creative expression, and the induction of ‘flow’ states. During handicraft-based activities, individuals engage in emotional exploration and cognitive articulation via unconscious linkages with their external environment, thereby promoting introspective reflection and heightened self-awareness. Embodied cognition elucidates the interplay between somatic physiological states and psychological functioning. It emphasizes the role of the body as a mediator of cognitive engagement with the environment, establishing a bidirectional relationship between mental processes and external context. As discussed earlier, creative endeavors can activate neural regions implicated in self-referential processing and emotional modulation, which are integral to psychological well-being and exemplify the mechanisms of embodied cognition (Zwir et al., 2022).

When employed therapeutically, handicraft-based artistic practices can stimulate cognitive engagement and facilitate affective expression in patients. Furthermore, the cognitive dimension encompasses the comprehension and aesthetic appreciation of the created artwork. This enables the creator to interpret the emotional and intentional underpinnings of their work from a personal standpoint (Damiano et al., 2023). The practice of handicraft art demands sustained attentional focus and immersion in the present moment; such concentrated engagement can foster a sense of bodily and creative integration, thereby enhancing self-efficacy and personal worth (Puente-Díaz et al., 2023). Consequently, structured training in handicraft art therapy may assist individuals with anxiety disorders in achieving emotional and cognitive alignment during the artistic process. Moreover, it offers a means to evaluate patients’ cognitive and emotional states through the distinctive qualities and interpretive depth of their artistic productions.

### 4.2 Social support and interpersonal relationships

Handicraft art therapy plays a pivotal role in enhancing social support and strengthening interpersonal relationships. Typically conducted in group or community environments, handicraft activities facilitate social interaction, which in turn fosters interpersonal connections and cultivates a supportive atmosphere. Studies indicate that social support substantially contributes to better mental health, especially in times of stress or emotional difficulty (Heniff et al., 2024). Through collaborative craft-making, individuals are able to share personal experiences and emotional states. This exchange aids in building more robust social bonds, reducing feelings of isolation, and increasing overall life satisfaction (Puente-Díaz et al., 2023). A study conducted among residents of retirement communities in California found that those who participated in handicraft leisure activities were better able to complete tasks involving abstract visual information and non-verbal reasoning, suggesting that handicraft leisure activities may help maintain cognitive function and that craft activities function through social support (Mashinchi et al., 2023). The above studies demonstrate that engaging in manual activities can foster a stronger sense of social belonging by facilitating cooperation and interpersonal communication, which in turn contributes positively to mental wellbeing.

### 4.3 Emotions and cognition

Emotion plays a crucial role in artistic creation, shaping not only the creative process but also deeply influencing the final expression of the work. Studies indicate that artistic creation often functions as a medium for conveying the artist’s emotional experiences. For instance, in her self-portraits, Frida Kahlo communicates physical suffering and emotional distress through her use of color and composition. This emotional articulation is closely connected to her personal life events. Quantitative assessments of Kahlo’s paintings reveal a significant association between her color selections and emotional states, with short-wavelength hues (such as red and yellow) frequently linked to experiences of pain and frustration. This suggests that emotion in artistic creation serves not only as a source of inspiration but also as an indicator of the emotional depth embedded within the artwork (Turkheimer et al., 2022). Consequently, considering artistic creation as a form of emotional recovery implies that art therapy can assist individuals in releasing adverse emotions and enhancing psychological well-being. Research demonstrates that children engaged in art therapy show notable progress in emotional articulation and self-understanding, underscoring the beneficial impact of emotion in creative practices (Loreskär and Binder, 2024).

Handicraft art functions as a therapeutic instrument that supports individuals in coping with negative emotions and developing psychological adaptability. An experimental study involving medical students found that learning visual arts and participating in creative activities enhanced their mindfulness, self-awareness, and ability to manage stress. These improvements contribute to their overall wellness, decrease burnout, and facilitate the effective processing and release of distressing emotions (Loreskär and Binder, 2024). Another investigation into the effects of handicraft training on mental health reported that, after 3 months, participants exhibited marked increases in self-efficacy and self-esteem (Latifian et al., 2024). This demonstrates that such training not only elevates self-regard but also cultivates greater psychological resilience, assisting individuals in reintegrating into social environments.

**Figure 3:**
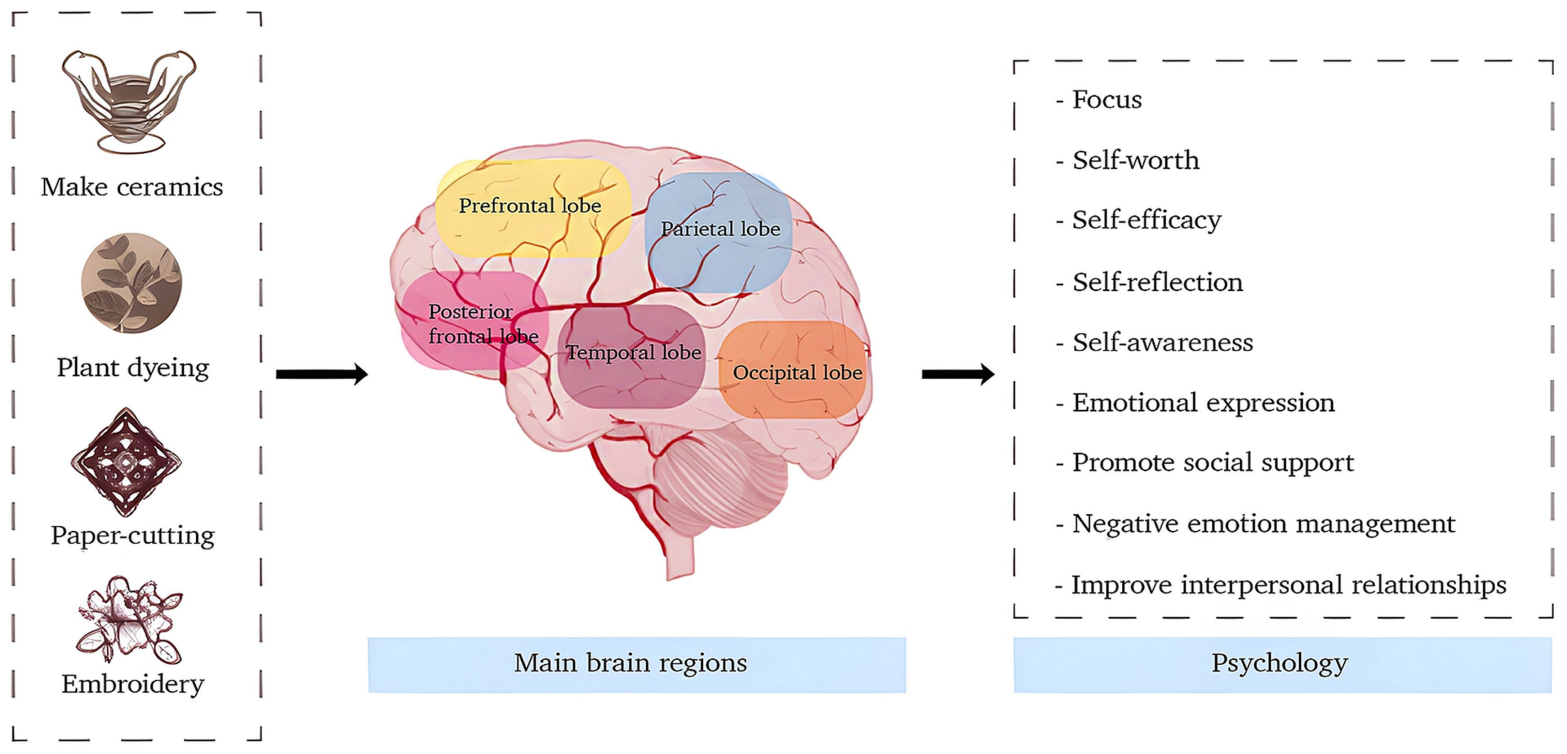
The psychological principles of handicraft art therapy in alleviating anxiety (original figure created by the authors).

In summary, handicraft art therapy, through the coordinated engagement of hand and brain, demonstrates psychological restorative effects. It activates the dynamic interplay between cognition and emotion via creative engagement. This process allows individuals to reconstruct their internal psychological landscape while simultaneously reshaping the external material world, as illustrated in Figure 3.

## 5 Practical applications in clinic of handicraft art therapy

The theoretical basis and clinical application of arts and handicrafts as occupational therapy interventions for anxiety are grounded in the fundamental tenets of occupational therapy, which employs “purposeful activities” to foster both physical and psychological well-being. In the context of anxiety management, occupational therapy facilitates the redirection of attention and the restoration of a sense of control and accomplishment through structured, achievable tasks—such as pottery, embroidery, knitting, or paper crafts—paralleling the behavioral activation component of cognitive behavioral therapy (CBT) (Saxena and Sahai, 2025; Choi and Kim, 2022). Practitioners utilize methods including activity analysis, individualized adaptation, and environmental adjustments to incorporate handicraft-based tasks into staged treatment plans, with the goal of strengthening patients’ self-efficacy (Müllenmeister et al., 2025). Evidence shows that professionally supervised handicraft interventions can lead to short-term improvements in anxiety, stress, depressive symptoms, self-efficacy, emotional state, and overall quality of life (Spee et al., 2025; DeGraba et al., 2020).

Literature research reveals that within the field of mental health, there is considerable variation regarding the target populations, types of interventions, key focus areas, and implementation methods when using handicrafts as therapeutic tools. For example, one study found that handicraft-based activities, which combine personalized and group occupational therapy approaches, lead to significant improvements in the cognitive functioning of individuals diagnosed with schizophrenia (Shimada et al., 2024). By engaging in these handicraft exercises, patients not only derive pleasure from creative self-expression but also experience a reduction in feelings of loneliness and anxiety through interaction with therapists and peers. Additional research indicates that regular participation in pottery-making can help alleviate symptoms of anxiety and enhance overall quality of life for those with anxiety disorders. Likewise, weaving activities have proven effective in assisting older adults in managing anxiety and depressive symptoms, resulting in notable enhancements in their psychological well-being and greater life satisfaction. A systematic review summarized various studies on the impact of handicraft interventions on mental health and well-being. These interventions, implemented by art therapists, nurses, or occupational therapists, took various forms, including pottery, embroidery, weaving, paper crafts, and woodworking. The review found that all studies reported positive improvements in the short term, including a reduction in anxiety symptoms (Bukhave et al., 2025).

**Figure 4:**
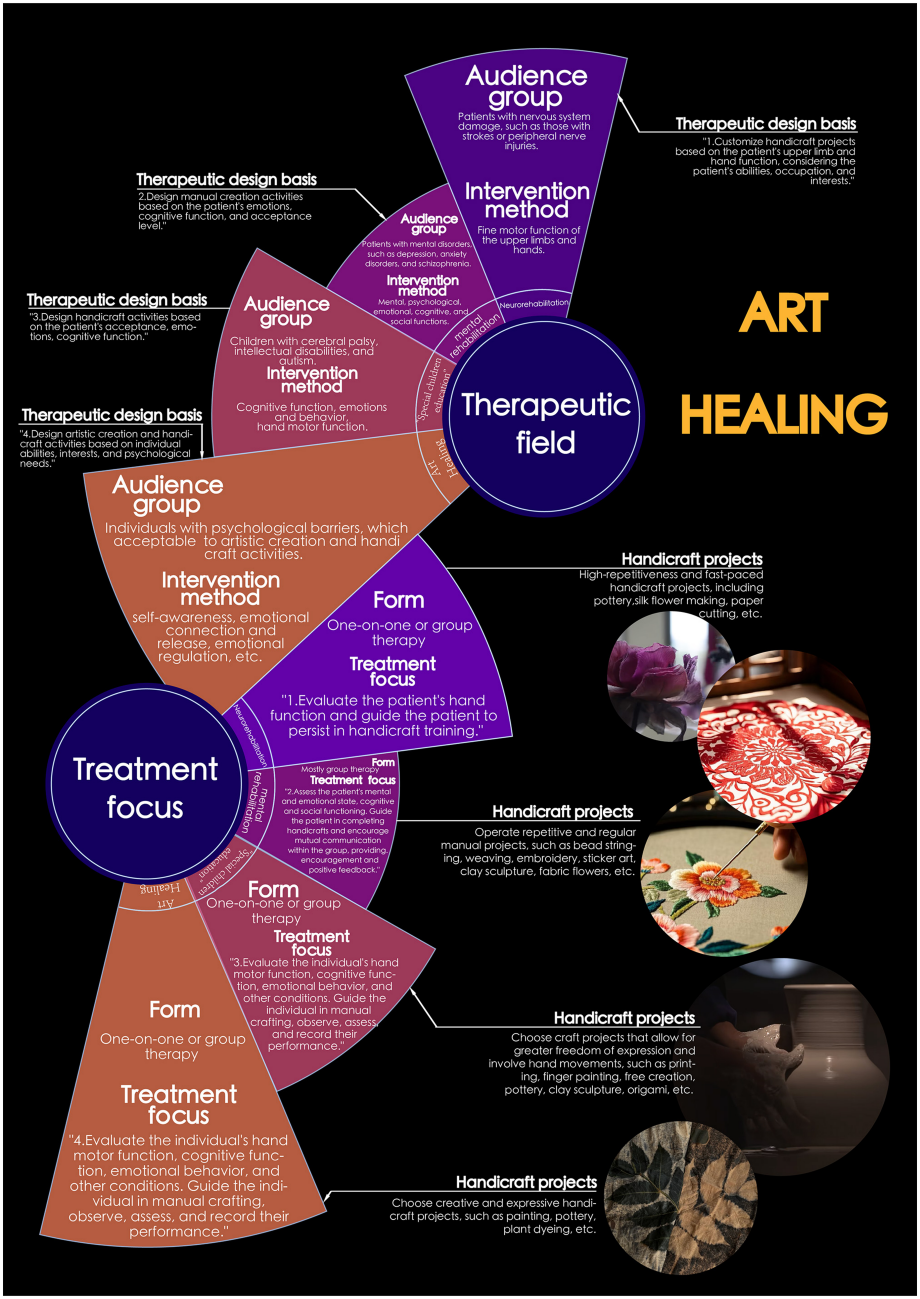
The visualization of the application of handicraft art therapy in clinical practice (original figure created by the authors).

The authors summarize common methods of applying handicraft therapy in clinical settings in Table 1 and offer an intuitive visualization in Figure 4.

**Table 1:** Common handicraft therapy in clinical applications (original table created by the authors).

**Treatment field**	**Target group**	**Intervention focus**	**Therapeutic schedule design**	**Handicraft projects**	**Treatment focus**	**Form**
Neurorehabilitation	Patients with nervous system damage, such as those with strokes or peripheral nerve injuries.	Fine motor function of the upper limbs and hands.	Customize handicraft projects based on the patient’s upper limb and hand function, considering the patient’s abilities, occupation, and interests.	High-repetitiveness and fast-paced handicraft projects, including pottery, silk flower making, paper cutting, etc.	Evaluate the patient’s hand function and guide the patient to persist in handicraft training.	One-on-one or group therapy
Mental rehabilitation	Patients with mental disorders, such as depression, anxiety disorders, and schizophrenia.	Mental, psychological, emotional, cognitive, and social functions.	Design manual creation activities based on the patient’s emotions, cognitive function, and acceptance level.	Operate repetitive and regular manual projects, such as bead stringing, weaving, embroidery, sticker art, clay sculpture, fabric flowers, etc.	Assess the patient’s mental and emotional state, cognitive and social functioning. Guide the patient in completing handicrafts and encourage mutual communication within the group, providing encouragement and positive feedback.	Mostly group therapy
Special children education	Children with cerebral palsy, intellectual disabilities, and autism.	Cognitive function, emotions and behavior, hand motor function.	Design handicraft activities based on the patient’s acceptance, emotions, cognitive function.	Choose craft projects that allow for greater freedom of expression and involve hand movements, such as printing, finger painting, free creation, pottery, clay sculpture, origami, etc.	Evaluate the individual’s hand motor function, cognitive function, emotional behavior, and other conditions. Guide the individual in manual crafting, observe, assess, and record their performance.	One-on-one or group therapy
Art healing	Individuals with psychological barriers, which acceptable to artistic creation and handicraft activities.	Self-awareness, emotional connection and release, emotional regulation, etc.	Design artistic creation and handicraft activities based on individual abilities, interests, and psychological needs.	Choose creative and expressive handicraft projects, such as painting, pottery, plant dyeing, etc.	Evaluate the individual’s hand motor function, cognitive function, emotional behavior, and other conditions. Guide the individual in manual crafting, observe, assess, and record their performance.	One-on-one or group therapy

## 6 Handicraft creation training and brain emotional reconstruction—‘hand-brain-emotion’ axis

The clinical management of anxiety disorders involves the integration of flexible and precise hand movements with complex neurocognitive processes in the brain. Fine motor functions primarily encompass hand dexterity, visual perception, and hand-eye coordination. From a sensorimotor perspective, the hand occupies a substantial projection area within the precentral and postcentral gyri of the cerebral cortex. The “hand-brain perception” theory in stroke rehabilitation proposes that when external stimuli are perceived, sensory information is transmitted via ascending pathways to specific brain control regions (Hazelton et al., 2022). The brain subsequently processes, analyzes, and integrates this information, which is then conveyed through descending pathways to relevant muscles, bones, and other peripheral effectors, ultimately expressed through hand movements and other actions. Drawing inspiration from this concept, the authors have extended neurorehabilitation principles to the treatment of mental disorders, introducing the “hand-brain-emotion” axis theory. This framework posits that, based on the abnormal brain regions and neuro-modulatory mechanisms associated with anxiety disorders, targeted peripheral interventions can facilitate remodeling of the central nervous system, thereby accelerating the alleviation of anxiety symptoms. In the following content, we will elaborate on how handicraft activities influence anxiety from a neurophysiological standpoint, specifically through the lens of the ‘hand-brain-emotion’ axis theory.

### 6.1 Regulation of the sensorimotor cortex and cognitive resources by fine motor activities

Engaging in precise manual tasks, such as embroidery or model assembly, requires intense focus on real-time sensorimotor feedback. This concentrated attention on the ongoing task consumes significant cognitive capacity, effectively disrupting neural circuits involved in anxiety-related intrusive thoughts and rumination (Wang et al., 2026). Neuroimaging studies confirm that fine hand movements strongly activate the brain’s sensorimotor cortical network. For example, research shows that in individuals with early-stage psychosis, functional connectivity patterns within the sensorimotor network are associated with clinical symptoms, including anxiety (Hoheisel et al., 2024; Zhao et al., 2022). Specifically, fine motor tasks engage areas such as the primary sensorimotor cortex, supplementary motor area, and parietal cortex. The coordinated activation across these regions may help suppress hyperactivity in the default mode network, whose excessive activity is directly related to anxious and worrisome thinking (Niechwiej-Szwedo et al., 2021). Thus, fine motor activities can achieve emotional regulation by effectively “preempting” the activity of this network.

Beyond immediate neural activation, sustained fine motor control also promotes neuroplasticity—the brain’s ability to reorganize in response to learning and experience. Studies indicate that combining specific tasks, such as virtual reality training, can enhance this plasticity in a targeted manner (Liu et al., 2024). Strengthening plasticity, particularly within the prefrontal cortex, is believed to improve its “top-down” regulatory control over limbic structures like the amygdala, a key neural basis for emotional regulation (Huang et al., 2025; Hoffman et al., 2021). In summary, fine motor activities reduce anxiety through multiple neural mechanisms: occupying cognitive resources, modulating key brain network dynamics, and fostering adaptive neuroplastic changes.

### 6.2 The role of bilateral coordinated stimulation in cerebral hemisphere integration and emotional balance

**Figure 5:**
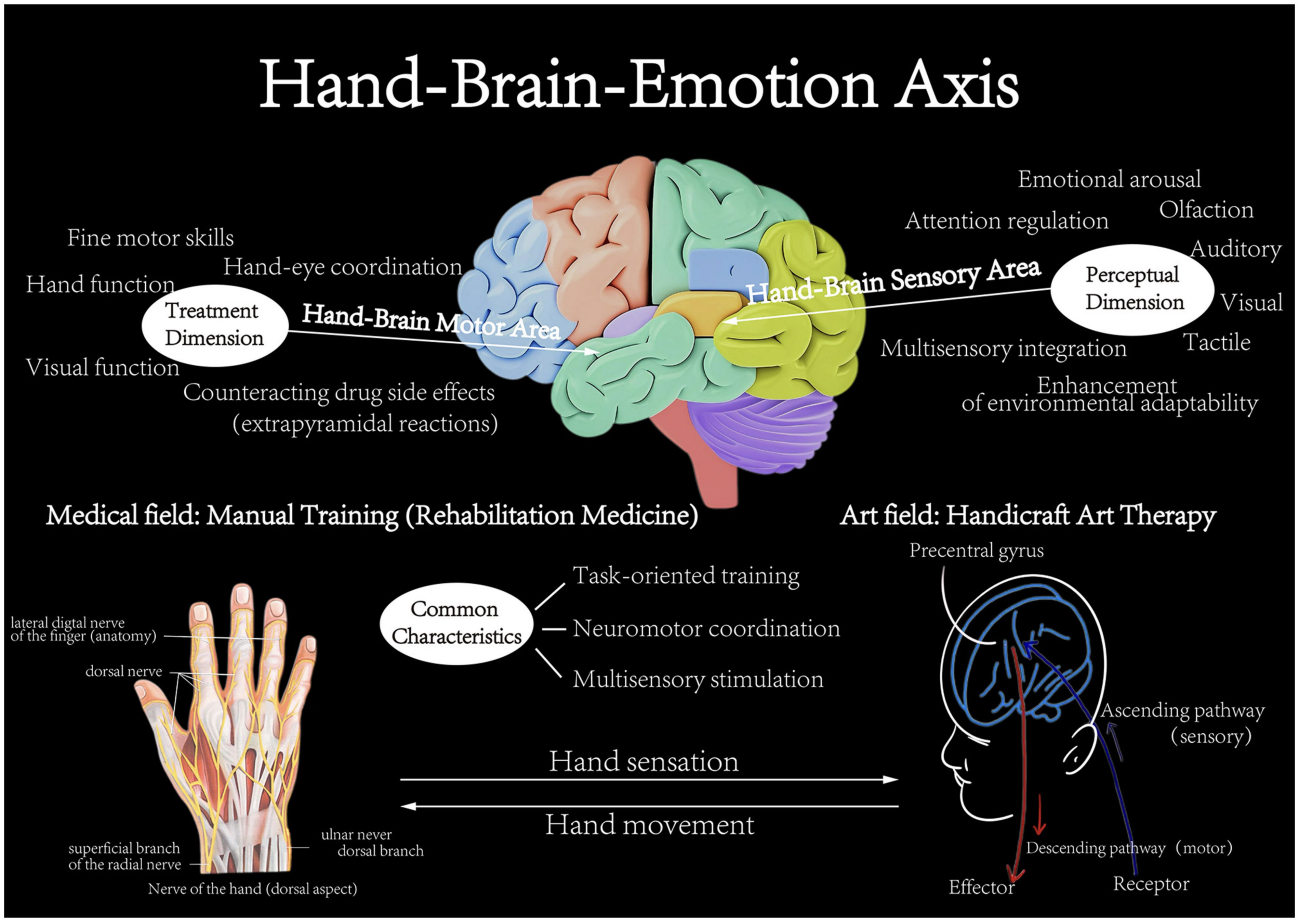
Schematic diagram of ‘hand-brain-emotion’ axis mechanism in handicraft rehabilitation training and the perceptual dimension (original figure created by the authors).

Many handicraft-based activities—including pottery wheel throwing and weaving—require the simultaneous use of both hands, generating bilateral and rhythmic sensory input. This form of bilateral coordinated engagement promotes information transfer and functional integration between the left and right cerebral hemispheres. From a neurophysiological perspective, such integration is likely supported by activation of the corpus callosum and related commissural fibers, which may help reduce functional asymmetries between the hemispheres (Andrulyte et al., 2024). Certain anxiety-related disorders have been associated with atypical lateralization of hemispheric functions; therefore, activities that improve interhemispheric coordination could aid in reestablishing emotional balance (Yao and Kendrick, 2022). Theoretical frameworks—including those drawing on the bilateral stimulation principle used in Eye Movement Desensitization and Reprocessing (EMDR) therapy—suggest that bilateral sensory input can promote adaptive information processing within limbic networks, thereby reducing emotional arousal linked to traumatic or anxiety-inducing memories (Stingl et al., 2025; Pape et al., 2024). While direct studies on the neural mechanisms of bilateral stimulation handicraft practices are still limited, existing research on related neural pathways provides support for this view. For example, brain regions involved in sensorimotor integration, such as the cerebellum, are now recognized to have roles beyond traditional motor coordination and to participate in regulating various emotional behaviors, including anxiety and stress responses (Rudolph et al., 2023). The cerebellum plays an important role in emotion regulation through its connections with limbic structures (da Silva et al., 2023). Furthermore, neural circuits that help maintain emotional balance—such as those in the ventral hippocampus—contain specialized microcircuits and long-range projections that process fear, anxiety, and reward-related information. The normal operation of these circuits relies on effective communication and integration across multiple brain regions (van der Veldt et al., 2025; Cai et al., 2023). Thus, bilateral coordinated activities in handicrafts may encourage adaptive information processing, decrease emotional arousal, and ultimately reduce anxiety while supporting emotional stability. These outcomes are likely accomplished by enhancing interhemispheric connectivity and modulating deeper brain networks involved in sensorimotor and emotional processing, such as the limbic-cerebellar circuitry. The schematic diagram of the ‘hand-brain-emotion’ axis mechanism is shown in Figure 5.

## 7 Discussions

Handicraft art has a profound influence on the ‘hand-brain-emotion’ axis. Its underlying mechanisms can be explained from the perspectives of neuroscience and cognitive development. This review emphasizes the role of handicraft training in strengthening hand function, thereby activating the brain’s physiological emotional link and forming multi-channel stimulation-feedback training. Customized handicraft operations, with therapeutic intent, can trigger physiological feedback mechanisms in the brain, enhancing emotional regulation, self-awareness, and self-confidence in patients. The interaction between handicraft creation and the brain reveals the complexity of human cognition and emotion, underscoring its importance in the healing process for patients with anxiety disorders.

Despite significant progress in studying “handicraft art creation” as a healing method and its neural mechanisms, there are still notable limitations and controversies. Integrating handicraft arts into occupational therapy requires therapists to accurately analyze activities and personalize them based on the patient’s anxiety type; disease severity; cultural background; interests; and physical function. Systematic reviews indicate that existing studies show significant differences in activity media, treatment duration, and theoretical foundations (Wang and Jiang, 2023). Moreover, interventions are often implemented by various professionals, which emphasizes the need for therapists to exercise professional judgment and make adjustments according to specific contexts (Bukhave et al., 2025; Tan et al., 2024). This heterogeneity means that a standardized approach is ineffective, and therapists must select activities that align with the patient’s personal goals. For example, for adolescents with functional movement disorders, interventions should integrate psychological support with sensorimotor activities to construct therapeutic meaning (Nilles et al., 2022).

The objective assessment of curative effects is a major challenge in practice. Self-report scales lack long-term follow-up data and standardized assessment tools for craft-based interventions (Bukhave et al., 2025; Gallegos et al., 2026). A future direction is translating neurophysiological indicators into clinical biofeedback tools, given that anxiety is associated with intolerance of uncertainty and involves complex neural circuits. Monitoring indicators such as heart rate variability (HRV) and electroencephalogram (EEG) may provide an objective means of understanding how interventions regulate autonomic balance and cortical communication. Furthermore, the quality of the treatment relationship, creating a supportive environment, and the co-construction of the meaning of activities are key contextual factors. For instance, handicraft art therapy can promote emotional processing in a safe and trusting social environment, but creating artwork in the presence of others may trigger feelings of vulnerability, and experiences of failure may make individuals with fragile personalities feel deficient (Huotilainen et al., 2018). Therefore, positive therapeutic outcomes are more likely to occur when individuals face difficulties or failures while receiving emotional support.

Additionally, many studies focus on small sample sizes or particular groups, such as art students, college students, and children with anxiety, which restricts the generalizability and applicability of the findings. Finally, while some studies have indicated links between handicraft art and psychological mechanisms like emotions and empathy, the exact neural basis of these connections still needs more investigation (Ardizzi et al., 2021).

Future studies should explore the role of “handicraft art creation” as a therapeutic intervention and its connection to brain neural mechanisms from an interdisciplinary standpoint. Cross-disciplinary research can reveal the neural pathways through which handicraft creation training influences individuals with anxiety disorders. Such insights are crucial for effectively managing anxiety-related conditions. For example, neuroscience research has revealed the role of extensive neural circuits, ranging from sensorimotor integration to reward evaluation, in anxiety (Mahima and Pentela, 2025; Yücel et al., 2017). Employing advanced neuroimaging methods, including functional near-infrared spectroscopy, allows for the monitoring of real-time brain responses during handicraft creation sessions, thereby deepening our comprehension of the associated emotional and cognitive shifts (Yücel et al., 2017). Psychological studies highlight the impact of factors like expectations and coping mechanisms on clinical outcomes (Furmark et al., 2025). By synthesizing insights across behavioral, cognitive, emotional, and neurological dimensions, more holistic theoretical frameworks can be established, paving the way for innovative, technology-supported intervention approaches. From a sociological standpoint, investigations should examine response variations across diverse populations—such as children, older adults in community settings, and distinct subtypes of anxiety disorders—to identify the optimal intervention “dosage” in terms of frequency, duration, and intensity, thereby contributing to evidence-based clinical protocols (Furmark et al., 2025). Interdisciplinary cooperation is essential for positioning handicraft-based activities as a viable approach for managing anxiety disorders, necessitating collaboration among occupational therapists, neuroscientists, psychologists, and art therapists. For instance, integrating technologies like virtual reality (VR) and brain-computer interfaces (BCI) can enable the creation of highly immersive and individualized handicraft simulation environments, while real-time neural activity monitoring offers novel avenues for understanding and harnessing neuroplasticity (Valente et al., 2026; Drigas and Sideraki, 2024). Such cross-disciplinary synergy will help advance art and handicraft-based interventions for anxiety from experiential applications toward precision-oriented therapeutic practice.

## 8 Conclusion

The author emphasizes in this review the importance of obtaining hand exercises from craft training, thereby activating the physiological emotional connections in the brain and forming multi-channel stimulus feedback training. Therapeutically customized handicraft art operations may stimulate the brain’s feedback mechanisms, improving patients’ emotional regulation, self-awareness, and confidence. The interaction between handicraft art activities and the brain illustrates the complexity of human cognition and emotion, emphasizing its important role in healing patients with anxiety disorders. Further study will begin with the specific design of handicraft art, progressing from simple hand training to integrative brain cognition, ultimately addressing mental and emotional health. Future research will conduct in-depth clinical practices to gather real feedback on the ‘hand-brain-emotion’ axis, providing scientific evidence for using craft art in treating anxiety disorders and fostering innovation in neuro-psychological health.

## References

[ref1] Andrulyte I. De Bezenac C. Branzi F. Forkel S. J. Taylor P. N. Keller S. S. (2024). The relationship between white matter architecture and language lateralization in the healthy brain. J. Neurosci. 44:e0166242024. doi: 10.1523/JNEUROSCI.0166-24.2024 39375038 PMC11638810

[ref9001] Ardizzi M. Ferroni F. Umiltà M. A. Pinardi C. Errante A. Ferri F. . (2021). Visceromotor roots of aesthetic evaluation of pain in art: an fMRI study. Soc. Cogn. Affect. Neurosci. 16, 1113–1122. doi: 10.1093/scan/nsab066 33988702 PMC8599194

[ref2] Barrett K. C. Barrett F. S. Jiradejvong P. Rankin S. K. Landau A. T. Limb C. J. (2020). Classical Creativity: A Functional Magnetic Resonance Imaging (fMRI) Investigation of Pianist and Improviser Gabriela Montero. NeuroImage. 209:116496. doi: 10.1016/j.neuroimage.2019.116496 31899286

[ref3] Birnie M. T. Kooiker C. L. Short A. K. Bolton J. L. Chen Y. Baram T. Z. (2020). Plasticity of the reward circuitry after early-life adversity: mechanisms and significance. Biol. Psychiatry 87, 875–884. doi: 10.1016/j.biopsych.2019.12.018, 32081365 PMC7211119

[ref4] Bukhave E. B. Creek J. Linstad A. K. Frandsen T. F. (2025). The effects of crafts-based interventions on mental health and well-being: a systematic review. Aust. Occup. Ther. J. 72:e70001. doi: 10.1111/1440-1630.70001, 39956657 PMC11830576

[ref5] Cai C. Y. Tao Y. Zhou Y. Yang D. Qin C. Bian X. L. . (2023). Nos1(+) and Nos1(−) excitatory neurons in the BLA regulate anxiety- and depression-related behaviors oppositely. J. Affect. Disord. 333, 181–192. doi: 10.1016/j.jad.2023.04.049, 37080493

[ref6] Choi S. Kim D. (2022). Effects of combining cognitive behavioral therapy with bilateral upper Limb training in stroke patients: a randomized controlled trial. Occup. Ther. Int. 2022, 1–9. doi: 10.1155/2022/4688113, 35912312 PMC9282985

[ref7] da Silva G. N. Seiffert N. Tovote P. (2023). Cerebellar contribution to the regulation of defensive states. Front. Syst. Neurosci. 17:1160083. doi: 10.3389/fnsys.2023.1160083, 37064160 PMC10102664

[ref8] Damiano C. Gayen P. Rezanejad M. Banerjee A. Banik G. Patnaik P. . (2023). Anger is red, sadness is blue: emotion depictions in abstract visual art by artists and non-artists. J. Vis. 23:1. doi: 10.1167/jov.23.4.1, 37010831 PMC10080919

[ref9] de Oliveira Lima C. A. da Silva Mendonça P. Crispim J. G. da Silva R. L. Silva R. C. de Melo Alcantara L. F. . (2026). Medial prefrontal cortex, dopamine and glutamate modulation in regulating reward-seeking and frustration responses. Neurosci. Lett. 874:138506. doi: 10.1016/j.neulet.2026.138506, 41520996

[ref10] DeGraba T. J. Williams K. Koffman R. Bell J. Pettit W. Kelly J. . (2020). Efficacy of an interdisciplinary intensive outpatient program in treating combat-related traumatic brain injury and psychological health conditions. Front. Neurol. 11:580182. doi: 10.3389/fneur.2020.580182, 33536993 PMC7848806

[ref11] Dieterich-Hartwell R. (2023). Art and movement as catalysts for insight into the human condition of depression. Psychiatr. Rehabil. J. 46. doi: 10.1037/prj0000550, 37548621

[ref12] Drigas A. Sideraki A. (2024). Brain neuroplasticity leveraging virtual reality and brain-computer Interface technologies. Sens. 24:5725. doi: 10.3390/s24175725. PMC11397861 39275636

[ref13] Feng Q. He L. Yang W. Zhang Y. Wu X. Qiu J. (2019). Verbal creativity is correlated with the dynamic reconfiguration of brain networks in the resting state. Front. Psychol. 10:894. doi: 10.3389/fpsyg.2019.00894, 31068873 PMC6491857

[ref14] Furmark T. Wahlstedt K. Faria V. (2025). Revisiting the SSRI vs. placebo debate in the treatment of social anxiety disorder: the role of expectancy effects, neural responsivity, and monoamine transporters. Front. Psychol. 16:1531725. doi: 10.3389/fpsyg.2025.1531725, 40420982 PMC12104218

[ref15] Gallegos C. Saul T. Timmerman R. A. Chu F. Bindler R. J. Billings C. (2026). EBP beliefs, competencies, implementation self-efficacy, and access to mentors in a large U.S. healthcare system: an updated assessment of where we are now. Worldviews Evid.-Based Nurs. 23:e70107. doi: 10.1111/wvn.70107 41656980

[ref16] Gorka S. M. Teppen T. Radoman M. Phan K. L. Pandey S. C. (2020). Human plasma BDNF is associated with amygdala-prefrontal cortex functional connectivity and problem drinking behaviors. Int. J. Neuropsychopharmacol. 23, 1–11. doi: 10.1093/ijnp/pyz057, 31722379 PMC7064048

[ref17] Hazelton C. McGill K. Campbell P. Todhunter-Brown A. Thomson K. Nicolson D. J. . (2022). Perceptual disorders after stroke: a scoping review of interventions. Stroke 53, 1772–1787. doi: 10.1161/STROKEAHA.121.035671, 35468001 PMC9022686

[ref18] Heniff L. France N. F. Mavhu W. Ramadan M. Nyamwanza O. Willis N. . (2024). Reported impact of creativity in the Wakakosha ('you're worth it') internal stigma intervention for young people living with HIV in Harare, Zimbabwe. PLOS Glob Public Health 4:e0003909. doi: 10.1371/journal.pgph.0003909, 39499711 PMC11537412

[ref19] Hoffman A. N. Watson S. Fanselow M. S. Hovda D. A. Giza C. (2021). Region-dependent modulation of neural plasticity in limbic structures early after traumatic brain injury. Neurotrauma Rep 2, 200–213. doi: 10.1089/neur.2020.0045, 33937912 PMC8086520

[ref20] Hoheisel L. Kambeitz-Ilankovic L. Wenzel J. Haas S. S. Antonucci L. A. Ruef A. . (2024). Alterations of functional connectivity dynamics in affective and psychotic disorders. Biol Psychiatry Cogn Neurosci Neuroimaging 9, 765–776. doi: 10.1016/j.bpsc.2024.02.013, 38461964

[ref21] Huang M. Q. Liang X. L. Lin X. T. Li W. H. Jiang Y. H. Geng F. (2025). Amygdala-dependent signaling pathways and regulating circuits in fear memory. Synapse 79:e70026. doi: 10.1002/syn.70026, 40761078

[ref22] Hughes A. N. (2021). Glial cells promote myelin formation and elimination. Front. Cell Dev. Biol. 9:661486. doi: 10.3389/fcell.2021.661486, 34046407 PMC8144722

[ref23] Huotilainen M. Rankanen M. Groth C. Seitamaa-Hakkarainen P. Mäkelä M. (2018). Why our brains love arts and crafts: Implications of creative practices on psychophysical well-being. FormAkademisk 11. doi: 10.7577/formakademisk.1908

[ref24] Hutson P. Jame H. (2024). Neuroplasticity and creativity: transformative potential of fibre arts for growth and well-being. In novel trends in mental health. Faculty Scholarship, 689.

[ref25] Hwang K. A. Hwang Y. J. Hwang I. G. Song J. Jun Kim Y. (2019). Low temperature-aged garlic extract suppresses psychological stress by modulation of stress hormones and oxidative stress response in brain. J. Chin. Med. Assoc. 82, 191–195. doi: 10.1097/JCMA.0000000000000028, 30908412

[ref26] Ikeda Y. Igarashi Y. Potel M. (2022). Podiy: a system for design and production of pouches by novices. IEEE Comput. Graph. Appl. 42, 81–88. doi: 10.1109/MCG.2022.3145129, 35417341

[ref27] Jin Z. Yin J. Pan Y. Zhang Y. Li Y. Xu X. . (2024). Teach a man to fish: hyper-brain evidence on scaffolding strategy enhancing creativity acquisition and transfer. NeuroImage 297:12. doi: 10.1016/j.neuroimage.2024.120757, 39067552

[ref28] Koenigs M. Grafman J. (2009). The functional neuroanatomy of depression: distinct roles for ventromedial and dorsolateral prefrontal cortex. Behav. Brain Res. 201, 239–243. doi: 10.1016/j.bbr.2009.03.004, 19428640 PMC2680780

[ref29] Latifian M. Aarabi M. A. Esmaeili S. Abdi K. Raheb G. (2024). The role of internet addiction and academic resilience in predicting the mental health of high school students in Tehran. BMC Psychiatry 24:420. doi: 10.1186/s12888-024-05853-6, 38834960 PMC11151473

[ref30] Leckey J. (2011). The therapeutic effectiveness of creative activities on mental well-being: a systematic review of the literature. J. Psychiatric Mental Health Nurs. 18, 501–509. doi: 10.1111/j.1365-2850.2011.01693.x, 21749556

[ref31] Lewandowska P. Jakubowska N. Hryniewicz N. Prusinowski R. Kossowski B. Brzezicka A. . (2022). Association between real-time strategy video game learning outcomes and pre-training brain white matter structure: preliminary study. Sci. Rep. 12:20741. doi: 10.1038/s41598-022-25099-0, 36456870 PMC9715544

[ref32] Liu Y. Lin R. Tian X. Wang J. Tao Y. Zhu N. (2024). Effects of VR task-oriented training combined with rTMS on balance function and brain plasticity in stroke patients: a randomized controlled trial study protocol. Trials 25:702. doi: 10.1186/s13063-024-08519-6, 39434192 PMC11492617

[ref33] Loreskär P. Binder P. E. (2024). Nothing less than a creative triumph: a study of children admitted to hospital for serious somatic illness or injury and their experience of art therapy. Front. Psychol. 15:1353507. doi: 10.3389/fpsyg.2024.1353507, 38558783 PMC10979798

[ref34] Mackey S. Petrides M. (2014). Architecture and morphology of the human ventromedial prefrontal cortex. Eur. J. Neurosci. 40, 2777–2796. doi: 10.1111/ejn.12654, 25123211

[ref35] Mahima M. A. Pentela B. (2025). Future directions in anxiolytic therapy: a comprehensive review of novel targets and strategies. Curr. Neurovasc. Res. 22, 115–136. doi: 10.2174/0115672026394052250808075022, 40849761

[ref37] Mashinchi G. M. Mcfarland C. P. Hall S. Strongin D. L. Williams G. A. Cotter K. A. (2023). Handicraft art leisure activities and cognitive reserve. Clin. Neuropsychol. 38, 683–714. doi: 10.1080/13854046.2023.2253993 37674299

[ref38] Müllenmeister C. Königs G. Heinemann S. Schröder D. Müller F. Hummers E. . (2025). Acceptability of telehealth-delivered occupational therapy among individuals with long COVID using the theoretical framework of acceptability: a qualitative study. Int. J. Telemed. Appl. 2025:8879520. doi: 10.1155/ijta/8879520, 41477577 PMC12752885

[ref39] Niechwiej-Szwedo E. Wu S. Nouredanesh M. Tung J. Christian L. W. (2021). Development of eye-hand coordination in typically developing children and adolescents assessed using a reach-to-grasp sequencing task. Hum. Mov. Sci. 80:102868. doi: 10.1016/j.humov.2021.102868, 34509902

[ref40] Nilles C. Pringsheim T. M. Martino D. (2022). The recent surge of functional movement disorders: social distress or greater awareness? Curr. Opin. Neurol. 35, 485–493. doi: 10.1097/WCO.0000000000001074 35787596

[ref41] O’Leary K. B. Khan J. S. (2024). Pharmacotherapy for anxiety disorders. Psychiatr. Clin. N. Am. 47, 689–709. doi: 10.1016/j.psc.2024.04.012 39505448

[ref42] Ongür D. Ferry A. T. Price J. L. (2003). Architectonic subdivision of the human orbital and medial prefrontal cortex. J. Comp. Neurol. 460, 425–449. doi: 10.1002/cne.10609 12692859

[ref43] Orui J. Shiraiwa K. Tazaki F. Inoue T. Ueda M. Ueno K. . (2024). Psychophysiological and interpersonal effects of parallel group crafting: a multimodal study using EEG and ECG. Sci. Rep. 14:17883. doi: 10.1038/s41598-024-68980-w, 39095523 PMC11297208

[ref44] Orwig W. Diez I. Bueichekú E. Vannini P. Beaty R. Sepulcre J. (2021). Cortical networks of creative ability trace gene expression profiles of synaptic plasticity in the human brain. Front. Hum. Neurosci. 15:694274. doi: 10.3389/fnhum.2021.694274, 34381343 PMC8350487

[ref45] Pape V. Sammer G. Hanewald B. Schäflein E. Rauschenbach F. Stingl M. (2024). Apples and oranges: PTSD patients and healthy individuals are not comparable in their subjective and physiological responding to emotion induction and bilateral stimulation. Front. Psychol. 15:1406180. doi: 10.3389/fpsyg.2024.1406180, 38933577 PMC11203994

[ref46] Penninx B. W. Pine D. S. Holmes E. A. Reif A. (2021). Anxiety disorders. Lancet 397, 914–927. doi: 10.1016/S0140-6736(21)00359-7, 33581801 PMC9248771

[ref47] Puente-Díaz R. Cavazos-Arroyo J. Puerta-Sierra L. (2023). Becoming self-aware of feelings and performance: the influence of creative potential, self-evaluations, and metacognitive feelings on creative mindsets. J. Intell 11:138. doi: 10.3390/jintelligence11070138 37504781 PMC10381300

[ref48] Ren J. Zhou Y. Luo J. (2020). fMRI data for creativity reconfigure new conceptual knowledge through hippocampus-middle temporal gyrus. Data Brief 30:105538. doi: 10.1016/j.dib.2020.105538, 32346572 PMC7182676

[ref49] Rudolph S. Badura A. Lutzu S. Pathak S. S. Thieme A. Verpeut J. L. . (2023). Cognitive-affective functions of the cerebellum. J. Neurosci. 43, 7554–7564. doi: 10.1523/JNEUROSCI.1451-23.2023, 37940582 PMC10634583

[ref50] Saxena K. Sahai A. (2025). Understanding the effectiveness of cognitive Behavioural therapy: a study on offenders. Ann. Neurosci. 32, 309–314. doi: 10.1177/09727531241288609, 39544670 PMC11559496

[ref51] Shimada T. Morimoto T. Nagayama H. Nakamura N. Aisu K. Kito A. . (2024). Effect of individualized occupational therapy on cognition among patients with schizophrenia: a randomized controlled trial. Schizophr. Res. 269, 18–27. doi: 10.1016/j.schres.2024.04.018, 38718691

[ref52] Shin L. M. Liberzon I. (2010). The Neurocircuitry of fear, stress, and anxiety disorders. Neuropsychopharmacology 35, 169–191. doi: 10.1038/npp.2009.83, 19625997 PMC3055419

[ref53] Shiraiwa K. Yamada S. Nishida Y. Toichi M. (2020). Changes in electroencephalography and cardiac autonomic function during craft activities: experimental evidence for the effectiveness of occupational therapy. Front. Hum. Neurosci. 14:621826. doi: 10.3389/fnhum.2020.621826, 33424571 PMC7793905

[ref54] Spee B. T. M. de Vries N. M. Zeggio S. Plijnaer M. Koksma J. J. Duits A. A. . (2025). Unleashing creativity in people with Parkinson's disease: a pilot study of a co-designed creative arts therapy. J. Neurol. 272:161. doi: 10.1007/s00415-024-12878-0, 39849173 PMC11758163

[ref55] Stingl M. Schäflein E. Spieler D. Henn M. Hanewald B. Sack M. (2025). Bilateral stimulation: differential effects in EEG and peripheral physiology. BJPsych Open 11:e278. doi: 10.1192/bjo.2025.10887, 41229305 PMC12641405

[ref56] Su W. Antonopoulos C. G. Valentini E. (2026). Network reorganisation reveals somato-motor transition from segregation to integration during tonic pain. Pain 165:1097. doi: 10.1097/j.pain.0000000000003897, 41553157

[ref57] Sylvester C. Myers M. Perino M. Sylvester C. M. Myers M. J. Perino M. T. . (2021). Neonatal brain response to deviant auditory stimuli and relation to maternal trait anxiety. Am. J. Psychiatry 178, 771–778. doi: 10.1176/appi.ajp.2020.20050672, 33900811 PMC8363512

[ref58] Tan M. Tang S. Feder S. Xiao J. Huang C. Cook A. . (2024). Interventions to promote readiness for advance care planning: a systematic review and meta-analysis. Int. J. Nurs. Stud. 156:104778. doi: 10.1016/j.ijnurstu.2024.104778, 38761437

[ref59] Tassone V. K. Nezhad F. G. Demchenko I. Rueda A. Bhat V. (2024). Amygdala biomarkers of treatment response in major depressive disorder: an fMRI systematic review of SSRI antidepressants. Psychiatry Res. Neuroimaging 338:111777. doi: 10.1016/j.pscychresns.2023.111777 38183847

[ref60] Teng F. Wang M. Lu Z. Zhang C. Xiao L. Chen Z. . (2024). Causal relationship between cortical structural changes and onset of anxiety disorder: evidence from Mendelian randomization. Cereb. Cortex 34. doi: 10.1093/cercor/bhae440, 39503246

[ref61] Turkheimer F. E. Liu J. Fagerholm E. D. Dazzan P. Loggia M. L. Bettelheim E. (2022). The art of pain: a quantitative color analysis of the self-portraits of Frida Kahlo. Front. Hum. Neurosci. 16:1000656. doi: 10.3389/fnhum.2022.1000656, 36118965 PMC9478482

[ref62] Valente M. Branco D. Bermúdez I. B. S. Fernandes J. C. Figueiredo P. Vourvopoulos A. (2026). EEG-based predictors of motor recovery during immersive VR-BCI rehabilitation. Sci. Rep. 16:7870. doi: 10.1038/s41598-026-39106-1 41663680 PMC12953618

[ref63] van der Veldt S. Bagot R. C. Ciocchi S. Ito R. Kheirbek M. Macaskill A. . (2025). Anxiety and beyond: diversity in ventral Hippocampus circuits and function. J. Neurosci. 45:e1304252025. doi: 10.1523/JNEUROSCI.1304-25.2025 41224652 PMC12614051

[ref64] Wang J. Chen C. Zhang X. Wang S. Hao R. Xu Y. . (2026). Symptom-specific neural circuits and corresponding transcriptomic profiles in depression. Behav. Brain Res. 500:115966. doi: 10.1016/j.bbr.2025.115966, 41308878

[ref65] Wang L. Jiang S. (2023). Effectiveness of parent-related interventions on cyberbullying among adolescents: a systematic review and Meta-analysis. Trauma Violence Abuse 24, 3678–3696. doi: 10.1177/15248380221137065, 36458864

[ref66] Wang H. R. Liu Z. Q. Nomi J. S. Schleifer C. H. Bearden C. E. Misic B. . (2025). Multivariate resting-state functional connectivity features linked to transdiagnostic psychopathology in early psychosis [Epub ahead of print]. doi: 10.1101/2025.06.04.654984, 41173200

[ref67] Won E. Kim Y. K. (2020). Neuroinflammation-associated alterations of the brain as potential neural biomarkers in anxiety disorders. Int. J. Mol. Sci. 21. doi: 10.3390/ijms21186546, 32906843 PMC7555994

[ref68] Xie M. Xiong Y. Wang H. (2024). The regulative role and mechanism of BNST in anxiety disorder. Front. Psych. 15:1437476. doi: 10.3389/fpsyt.2024.1437476, 39698215 PMC11652476

[ref69] Xie S. Zhang X. Cheng W. Yang Z. (2021). Adolescent anxiety disorders and the developing brain: comparing neuroimaging findings in adolescents and adults. Gen Psychiatr. 34, 23–31. doi: 10.1136/gpsych-2020-100411 PMC8340272 34423252

[ref70] Yao S. Kendrick K. M. (2022). Reduced homotopic interhemispheric connectivity in psychiatric disorders: evidence for both transdiagnostic and disorder specific features. Psychoradiology 2, 129–145. doi: 10.1093/psyrad/kkac016, 38665271 PMC11003433

[ref71] Yücel M. A. Selb J. J. Huppert T. J. Franceschini M. A. Boas D. A. (2017). Functional near infrared spectroscopy: enabling routine functional brain imaging. Curr Opin Biomed Eng 4, 78–86. doi: 10.1016/j.cobme.2017.09.011, 29457144 PMC5810962

[ref72] Zhao L. Bo Q. Zhang Z. Chen Z. Wang Y. Zhang D. . (2022). Altered dynamic functional connectivity in early psychosis between the salience network and visual network. Neuroscience 491, 166–175. doi: 10.1016/j.neuroscience.2022.04.002, 35398505

[ref73] Zhu J. Anderson C. Ohashi K. Khan A. Teicher M. H. (2023). Potential sensitive period effects of maltreatment on amygdala, hippocampal and cortical response to threat. Mol. Psychiatry 28:5128. doi: 10.1038/s41380-023-02002-5, 36869224 PMC10475146

[ref74] Zwir I. Del-Val C. Hintsanen M. Cloninger K. M. Romero-Zaliz R. Mesa A. . (2022). Evolution of genetic networks for human creativity. Mol. Psychiatry 27, 354–376. doi: 10.1038/s41380-021-01097-y, 33879864 PMC8960414

